# Tyrosinase Inhibitory Ability and In Vitro, In Vivo Acute Oral and In Silico Toxicity Evaluation of Extracts Obtained from Algerian Fir (*Abiesnumidica* de Lannoy ex CARRIERE) Needles

**DOI:** 10.3390/plants11182389

**Published:** 2022-09-14

**Authors:** Djamila Benouchenne, Ines Bellil, Sana Hazar Tachour, Salah Akkal, Hanène Djeghim, Fethi Farouk Kebaili, Gema Nieto, Douadi Khelifi

**Affiliations:** 1 Laboratoire de Génétique Biochimie et Biotechnologies Végétales, Faculté des Sciences de la Natureet de la Vie, Université Frères Mentouri Constantine 1, Constantine 25000, Algeria; 2 Département de Pathologieanatomique, Hopital Ben-Badis Constantine, Constantine 25000, Algeria; 3 Laboratory of Phytochemistry, Natural Products and Organic Synthesis (Physynor), Department of Chemistry, Faculty of Exact Sciences, University Brother Mentouri Constantine 1, Constantine 25000, Algeria; 4 Laboratoire de Biochimie, Biotechnologie et Division Santé, Centre de Recherche enBiotechnologie, Constantine 25000, Algeria; 5 Laboratoire de Génie Microbiologique et Applications, Département de Biochimie et de Biologie Moléculaire et Cellulaire, Faculté des Sciences de la Nature et de la Vie, Université Fréres Mentouri Constantine 1, Constantine 25017, Algeria; 6 Department of food Technology, Food Science and Nutrition, Faculty of Veterinary Sciences, Regional Campus of International Excellence “Campus Mare Nostrum”, University of Murcia, Espinardo, 30071 Murcia, Spain; 7 Ecole Nationale Supérieure de Biotechnologie, Constantine 25000, Algeria

**Keywords:** *A. numidica* leaves, Tyrosinase, toxicity, in vitro, in vivo, in silico, biochemical parameters, histopathological examinations

## Abstract

This study was designed to evaluate the tyrosinase inhibitory effect, in vitro, in vivo, and in silico toxicity of fractions isolated from *A. numidica* de Lannoy needles. The cytotoxicity of extracts was examined against *Artemia salina* larvae, while the toxicity of these extracts was tested by acute oral toxicity in mice; by administration of a dose of 2000 mg/kg b.w *A. numidica* leaves extracts. The blood samples were collected from the eye orbital sinus for further analysis of biochemical parameters. The absorption, distribution, metabolism, elimination, and toxicity (ADMET) properties were identified by the pkCSM web server. The data stated that ethyl acetate (EA) presented strong anti-tyrosinase apt. The results reported that ethyl acetate extract exhibited a strong inhibitory capacity against *A. salina* larvae with LD_50_ of 75.004 µg/mL. The data also showed that no mortality occurred, and no toxicity symptoms were observed in mice. The biochemical parameters revealed that both extracts significantly affected the hepatic profile by increasing ALT, AST, and alkaline phosphatase. Histopathological tests also confirmed that both fractions were toxic at this concentration on hepatic and renal tissues, with necrosis observed. The toxicity of molecules in silico revealed no effect on all examined biomolecules.It can be concluded that this plant was toxic on the liver and renal profiles and tissues at the dose studied.

## 1. Introduction

The appearance of several diseases, such as cancer, diabetes, cardiovascular, obesity, and hypertension, can be linked directly to the quality of current lifestyle [1,2]. Many studies have been undertaken to assess factors triggering these diseases, and they related these to the stress and the quality of food consumed, whether plants or of animal origin [3,4]. These diseases have become common phenomena, and they affect different races and all categories of society [5].

According to ethnobotanical research, natural substances from plants allowed civilization to survive deadly diseases. Hence, several and different bioactive molecules have been isolated and used as remedies of some health disorders [6,7,8,9]. Therefore, attention has recently turned to seek for effective natural drugs from plants including polyphenols as flavonoids and tannins [10,11,12]. 

In recent times, there has been a huge interest directed to plants, moving from popular medicine and appealing to nature. Plants have been intensely researched for their possible biological activities to stop the propagation of diseases as antioxidant agents or inhibitors of enzymes involved in pathologies. Following this axis, our attention is directed to an endemic essence of Algerian fir (*Abies numidica* de Lannoy ex Carrière) because there are no reports about the toxicity and enzyme inhibitory ability of this plant while it is used in traditional medicine. 

The Algerian fir is an endemic tree belonging to the Pinaceae family. It was used in traditional medicine to treat inflammation and respiratory problems, but this plant has scarcely been studied and little is known about it. In 2013, [13] stated the the GC-MS analysis of essential oil extracted from needles of Algerian fir collected from Sraidi region, Annaba (Algeria), and the results revealed that this oil was rich in bornyl acetate, camphene, alpha-pinene, and beta-pinene.Ramdani et al., [14] specifiedthe antimicrobial effect of essential oil obtained from needles obtained from Babors mounts, Setif, Algeria.In 2016, [15] reported the chemical composition and antimicrobial power of fractions extracted from *A. numidica* de Lannoy leaves, collected from Baborsmonts. Belhadj Mostefa et al. [16] specified diterpenes from *A. numidica* de Lannoy ex Carrière cones. Benouchenne et al. [17] described that ethyl acetate fraction extracted from *A. numidica* de Lannoy ex Carrière leaves was rich in phenolic compounds and presented potential antioxidant and antibacterial effects collected from Constantine City. In 2021, [18] designed the chemical composition of *n*-butanol fraction obtained from *A. numidica* leaves and this extract exhibited strong biological activities. Benouchenne et al. [19] reported the extraction of essential oilfrom Algerian fir needles, its GC/MS analysis, as well as its antioxidant and enzyme inhibitory ability, and the results disclosed potential alpha glucosidase inhibitory effects. 

The objectives of this research were to evaluate the ability of extracts to inhibit tyrosinase and to studytheir toxicity against *Artemia salina* larvae, and to assess the toxic effect of fractions of this plant in vivo (in mice) and in silico.

## 2. Results

### 2.1. Tyrosinase Inhibitory Ability 

**Figure 1:**
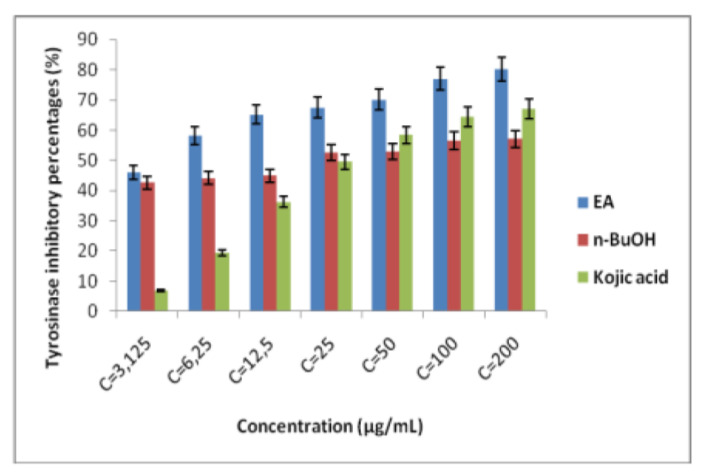
Tyrosinase inhibitory effect of EA, *n*-BuOH fractions and kojic acid at different concentrations.

The tyrosinase inhibitory potential of *A. numidica* de Lannoy ex Carrière extracts was examined. L-DOPA was used as a substrate for this experiment. Enzyme reaction was performed with tyrosinase, substrate and inhibitors (Extracts). Both extracts demonstrated a concentration-dependent inhibition. The results obtained proved that the two extracts exhibited a strong tyrosinase inhibitory activity in comparison with the standard (kojic acid) (Figure 1). The ethyl acetate fraction had a significant inhibitory power with IC at 50% of 3.72 ± 1.04 µg/mL, which is eighttimes lower than that of kojic acid (IC_50_ = 25.23 ± 0.78 µg/mL), while the *n*-butanol (*n*-BuOH) fraction had an inhibition concentration at 50% of 20.42 ± 0.98 µg/mL, which is also lower than that of the standard.

### 2.2. In Vitro, In Vivo and In Silico Toxicity of Extracts (EA and n-BuOH)

#### 2.2.1. Brine Shrimp Lethality Test

**Figure 2:**
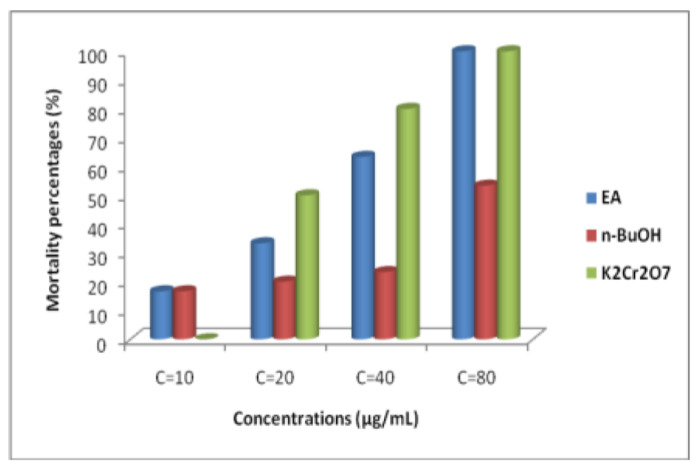
Cytotoxic effect of EA, *n*-BuOH fractions and standard (K_2_Cr_2_O_7_) on *A. salina* larvae.

In this work, the cytotoxicity of EA and *n*-BuOH extracts obtained from Algerian fir needles was tested, using *Artemia salina* larvae. Results illustrated by Figure 2 demonstrate that the EA fraction presented a strong cytotoxicity effect where LD_50_ = 56.66 µg/mL, compared with *n*-BuOH fraction which showed LD_50_ = 75.004 µg/mL, while dichromate potassium (K_2_Cr_2_O_7_) disclosed a LD_50_ of 20.09 µg/mL.

According to the report of [20], plant extracts with a lethal dose (DL_50_) < 1000 µg/mL, were regarded as toxic, while products with a DL_50_ > 1000 µg/mL were considered nontoxic. So, according to our findings, both extracts were toxic against *A. salina*.

#### 2.2.2. In Vivo Oral Acute Toxicity

- Behaviors, toxicity signs, and body weight variations

Table 1 illustrates the various behaviors observed and registered during 14 days of the oral acute toxicity test, after the treatment of the mice by *A. numidica* de Lannoy needles extracts. The results disclosed no mortality and no sign of toxicity at the concentration of 2000 mg/kg.

Table 2 represents the body weight change sets of control mice received physiological water, and mice administered by the extracts examined. According to the results, no significant effect occurred in mice body weights.

- Biochemical parameters
  - Effect of extracts (EA and *n*-BuOH) on renal function

Table 3 showed the creatinine and urea concentrations of serum after the treatment by 2000 mg/kg of both extract (EA and *n*-BuOH). From the results, there was a significant increase in serum creatinine in mice treated with EA which arrived at 3.1 mg/L, and to 4.4 mg/L in mice treated with *n*-BuOH, on the other hand. The creatinine level in the control mice is 2.4 mg/L. In contrast, no change in urea content in the three groups was recorded.

- Effect of EA and *n*-BuOH on hepatic parameters

The liver function examination consists of tests that make it possible to detect, diagnose or monitor certain pathologies such as hepatitis and damage caused by infections or toxic substances. The results obtained from the biochemical analysis of the hepatic parameters are summarised in Table 4, which showed a significant increase in transferases (AST and ALT) and alkaline phosphatase. On the other hand, no effect was observed for direct bilirubin, bilirubin, and Gamma-Glutamyl-Transferase.

- Effect of the extracts (EA and *n*-BuOH) on lipid profile

The results in Table 5 disclose that there were no significant changes in the lipid profile (triglyceride and cholesterol) after treatment with the two extracts compared with the control group.

- Effect of EA and *n*-BuOH extracts on organs tissues

The results obtained from histopathological sections of organs (liver and kidneys) after treatment with AE and *n*-BuOH extract as well as the control group of mice are presented in Table 6.

#### 2.2.3. ADMET Properties of Molecules Identified in *A. numidica* Leaves Extracts

According to [17,18], 12 molecules were identified by LC-MS/MS analysis in the ethyl acetate fraction, eightof which were also present in the fraction *n*-butanol, as shown in Table 7. In this study, these molecules were taken in order to determine and predict in silico their pharmacokinetic properties, in particular absorption, distribution, metabolism, clearance, and toxicity.

##### Physico-Chemical Properties of Molecules

The pKCSM server was used for the prediction of the physicochemical properties of twelve phenolic compounds identified by chromatographic analysis (LC-MS/MS) in the leaves of *A. numidica* de Lannoy ex Carrière. The results obtained are reported in Table 8. 

Dr Lipinski sets five rules to identify the molecules that can be used to designate a drug [21]. According to Lipinski’s rules of five, which reported that molecules suitable as a drug need to have no more than fivehydrogen bond donors, no more than 10 hydrogen bond acceptors, and a molecular weight less than 500 Daltons. Moreover, a coefficient logP not greaterthan 5 as well as molecules presenting a high bioavailability could be proposed as a drug likeness agent. Most of the molecules revealed a molecular weight between 154 and 448 daltons, however two molecules of them were above the maximum value determined by the rule, hesperidin and rutin (610 daltons) as shown in Table 8.

According to the results, for the log P parameter, all molecules had a log P value of less than 5, which indicated their hydrophilic character, but with different degrees. The compounds luteolin and apigenin were moderately soluble with increasing log P value, from 2.2824 to 2.5768; respectively. On the other hand, the molecules: rutin (−1.6871), hesperidin (−1.1566), chlorogenic acid (−0.6459), hyperoside (−0.5389), astragalin (−0.2445), luteolin-7-glucoside (−0.2445), apigetrin (0.0499), quercitrin (0.4887), protocatechic acid (0.796), and quercetin (1.9880) demonstrated a high solubility in aqueous media and recorded very low log P values, which attest tothe hydrophilic nature of these compounds (Table 8).

Six molecules obeyed Lipinski’s rules, these molecules have a hydrogen acceptor number less than 10, whereas hesperidin, astragalin, luteolin, hyperoside, rutin, and quercitrin have a hydrogen acceptor number greater than 10. Six molecules also have a hydrogen donor number greater than 5, namelyastragalin, luteolin-7-glucoside, hyperoside, rutin, apigetrin, and quercitin.

##### Pharmacokinetic Properties

The results of the prediction of the pharmacokinetic properties of the phenolic compounds of *A. numidica* deLannoy obtained using the pkCSM server are displayed in Table 8.

##### Absorption

In most cases, the main factors determining the oral bioavailability of the drug are probably metabolism and absorption at the intestinal level. The reduction in the polar surface is better correlated with the increase in the rate of permeation, and the increase in the number of rotary bonds has a negative effect on the rate of permeation [22].

The intestine is the primary site of absorption for orally administered drugs. The in-silico method is used to predict the proportions of molecules absorbed in the small intestine of humans. According to [23] molecules with an absorption percentage of less than 30% are less absorbed. The results disclosed that all these phenolic compounds are characterized by high intestinal absorption. A strong absorption of these molecules by the intestinal membrane makes possible their access to the blood, unlike the molecule: rutin which presented a weak intestinal absorption with an absorption percentage of 23.446%.

The skin is another organ where absorption can take place, and it is analyzed in the pharmaceutical and cosmetic fields to find products with a protective effect. This study is carried out in order to predict the effectiveness of plant-based molecules in the protection of the dermis. According to [23], constituents with a log kp < −2.5 have low permeability via the skin. The results proved that all the constituents have a strong permeability with a log kp = −2.735. While protocatechic acid demonstrated a value of log kp = −2.727.

##### Distribution

- Volume of Distribution (DV)

The volume of distribution (DV) is defined as the theoretical volume that the total drug’s dose is distributed in the blood plasma at the same concentration. When the DV of the drug is higher, it is well distributed into tissues. It can be affected by kidney failure or dehydration. This predictive model was constructed using the volume of distribution at steady state calculated in humans from 670 drugs.

According to [23] a molecule which had a DV < 0.71 L/kg (log DV < −0.15) had a low DV, while a product which has a DV > 2.81 L/kg (log DV > 0.45), has a high DV. The results displayed in Table 8 show that protocatechic acid had a low DV (log DV = −1.928). These results also revealed that rutin, quercetin, and quercitrin have a high DV in comparison with other molecules (log DV = 1.663, 1.559 and 1.517; respectively). Other compounds have an average DV.

- Permeability of the blood-brain barrier (BBB)

The brain is protected from exogenous molecules by a so-called blood-brain barrier. The ability of a drug to pass to the brain is a very important parameter, which helps to reduce the side effects and toxicity of a drug. The permeability of the blood–brain barrier is calculated in vivo on an animal model, it is expressed by log BB, it is the logarithmic ratio of the concentration of a drug in the brain and the plasma. According to [23], a product with a log BB > 0.3 can pass the BBB barrier, while a product with a log BB <−1 is poorly distributed in the brain. The results of this work are summarized in Table 8, showing that all the molecules tested have a low power to cross the BBB barrier.

##### Metabolism

The activity of cytochromes (CYP) can be changed under the action of inhibitory substances, which will cause a reduction in metabolism and therefore an increase in drug concentrations. It appears from our results (Table 8) that the compounds: hesperidin, protocatechic acid, chlorogenic acid, luteolin-7-glycoside, hyperoside, rutin, apigetrin, astragalin, and quercetrin have no inhibitory power on CYP isoforms and are normally metabolized. On the other hand, the three molecules (quercetin, luteolin, and apigetrin) that showed an inhibitory effect on three isoforms:

- Quercetin is a CYP1A2 inhibitor
- Luteolin is a CYP1A2 and CYP2C9 inhibitor
- Apigetrin is an inhibitor of CYP1A2 and CYP2CA9

##### Clearance

- Total clearance

Several physiological parameters make it possible to estimate the elimination of a xenobiotic. One of the most important is total clearance, which corresponds to the body’s ability to eliminate the molecule after returning to the bloodstream. Total clearance represents the volume of plasma purified per hour via the various organs involved in the elimination phase. Total clearance is determined by adding renal and hepatic clearances. The results stated a total clearance of arranged molecules from 0.211 to 0.566, while rutin presented a negative value of −0.369.

- Organic Cation Transporter (OCT2) Substrates

OCT2 plays a crucial role in the renal excretion of drugs and endogenous products. The results displayed in Table 8 indicate that no molecule could be a substrate for this transporter.

##### Toxicity

The toxicity profile of our phenolic compounds from the leaves of *A. numidica* de Lannoy from pkCSM server is presented in Table 8. Two parameters are taken to predict the toxicity of molecules in silico:

- Hepatotoxicity

Hepatotoxicity is also an important parameter for assessing the toxicity of a substance. The results obtained indicated that all the molecules examined are not toxic to the liver.

##### Maximum Tolerated Dose (MTD)

The maximum tolerated dose is the dose administered from which toxic effects are observed, but do not affect the vital functions of the animals. Pires et al. [23] reported that the maximum tolerated dose is considered low when MTD is equal to or less than 0.447 log (mg/kg/day), and high if MTD is greater than 0.447 log (mg/kg/day). According to the results presented in Table 8 part a, chlorogenic acid has a very low dose (log mg/kg/day = −0.134), followed by apigetrin (log mg/kg/day = 0.328). Rutin has a low dose (log mg/kg/day = 0.452). The remaining molecules presented a high dose, which variedfrom 0.495 to 0.814 log mg/kg/day.

## 3. Discussion

Tyrosinase is a copper metalloenzyme.It plays a crucial role in the production of melanin pigments. Inhibition of this enzyme is an effective method in regulating melanin production [24]. Melanin is a pigment synthesized in the melanocytes of the skin of living beings. The production of melanin is a physiological response for the protection of the skin against the harmful deterioration of ultraviolet radiation. However, the excessive production and accumulation of the latter in the skin results in diseases linked to hyperpigmentation, such as: freckles. Therefore, the regulation of synthesis is very important [25,26]. Currently, arbutin, kojic acid, and derivatives of hydroxy-quinones are widely used as synthetic drugs for the treatment of hyperpigmentation via the inhibition of tyrosinase. However, these chemicals can provoke harmful side effects.

With regard to finding tyrosinase inhibitor molecules, a massive number of studies and scientific researchers have been directed towards the use of herbal medicines. In order to discover active ingredients used in cosmetic preparations, our interest was directed towards an endemic plant, *A. numidica* de Lannoy. To our knowledge, few studies have been reported on the inhibitory effect of extracts from the leaves of this plant. Kojic acid is used as a standard. The results obtained proved that the two extracts exhibited a strong tyrosinase inhibitory activity in comparison with the standard. The ethyl acetate fraction had a significant inhibitory power with IC at 50% of 3.72 ± 1.04 µg/mL, which is eight times lower than that of kojic acid (IC_50_ = 25.23 ± 0.78 µg/mL), while the *n*-BuOH fraction had an inhibition concentration of 20.42 ± 0.98 µg/mL, which is also lower than that of the standard. Yang et al. [27] reported that pro-anthocyanidins extracted from *Pinus thunbergii* needles have a 50% inhibitory concentration of 37.64 μg/mL, and this value is higher than the value presented by the kojic acid standard (IC_50_ = 3.72 μg/mL). In this case, the extract has a low activity compared to the standard. Li et al. [28] found the potential effect of hyperoside against the enzyme tyrosinase. The inhibitory effect of this enzyme is probably due to the presence of secondary metabolites, such as phenolic compounds and flavonoids. Flavonoids have the ability to chelate metals, e.g., iron, copper, and silver, because of their structures, poly-hydroxylic [29]. In addition, flavonoids possessing a C-3’ hydroxyl group of the B ring, a C-3 hydroxyl group and a C-4 carbonyl group of the C ring increase the effects of tyrosinase inhibitors [30]. Nguyen et al. [31] reported that the presence of methoxyl and hydroxyl groups in the backbone of flavonoids plays a very important role in tyrosinase inhibition. In the same framework, Zolghadri et al. [26] stated that most of phenolic acids and flavonoids (Flavanoles, Flavan-3,4-diols, Flavanones) could inhibit competitively the tyrosinase enzyme. 

The brine shrimp lethality test is a suitable technique and often used to detect general toxicity of chemicals, screening for teratogenicity of drugs, ecotoxicology of biological organisms [32], and various pharmacological actions. The correlation between the brine shrimp lethality test and the inhibition of in vitro growth of human tumor cell lines showed the value of this biological test as a screening tool for anti-tumor drug research [33,34]. This approach may be useful for other biologically active compounds whose brine shrimp respond similarly to mammalian systemcorrespondents. For example, it has been shown that RNA-dependent polymerases of the DNA of *A. salina* are similar to the mammalian type and that the body has an ATPase dependenceon Na^+^ and K^+^ sensitive to ouabain, suchthat the compounds or extracts acting on these systems should be detected in this assay [35]. 

In this work, the cytotoxicity of extracts from the leaves of *A. numidica* de Lannoy ex Carrière has been tested using the *A. salina* larvae test. The results showed a significant inhibitory activity, where the LD_50_ presented by the extract EA is 56.66 µg/mL, and the LD_50_ disclosed by the *n*-BuOH fraction is 75 µg/mL, while potassium dichromate (K_2_Cr_2_O_7_) has an LD_50_ of 20.09 µg/mL.

So, according to the obtained results, the EA and *n*-BuOH extracts are toxic to *A. salina*. The current study has shown that *A. numdica* presented a cytotoxic effect, suggesting the presence of potential bioactive chemical components in the plant extract. As mentioned, plants produce a large number of natural products as secondary metabolites that have many and various pharmacological activities. The phytochemical screening of *A. numdica* leaves extracts revealed that the extracts contained high amounts of flavonoids, tannins, and saponins. These compounds have been implicated in cytotoxic activity [36]. It is possible that a wide range of structurally diverse phenolic compounds contribute to the overall pharmacological activity of the extract and synergistic effects between the principle’s assets may exist [37,38,39,40,41,42,43,44]. Our results are in the line of those reported by [45], which disclosed the potential cytotoxic effect of conifer resins against human epithelial and fibroblast cells.

Acute oral toxicity is the first step that must be tested on a product, if we know little information on its toxicity. Determining the LD_50_ in addition to recording the general behaviors of animals is one of the critical parameters for the assessment of early toxicity signs [46]. The present study illustrated that oral administration of 2000 mg/kg extracts of *A. numidica* did not show any effects on body weight or food consumption and did not cause any behavior changes and the LD_50_ is greater than 2000 mg/kg. 

To complete this study, the hepatic parameters (ALT, AST, GGT, PAL, total bilirubin, and direct bilirubin), lipid parameters (triglycerides, cholesterol), and renal parameters (creatinine and urea) were assessed. The results showed a significant increase in the concentrations of liver enzymes, especially AST, ALT, and PAL. The extracts also affected the concentration of creatinine. Ethyl acetate fraction increased the concentration of triglycerides while the *n*-BuOH fraction decreased after 14 days of toxicity testing. The damage to liver cells increases permeability of cell membranes, which results in a high release of aminotransferases in serum [47]. PAL rate is increased during cholestasis or bone damage. Cholestasis can be intrahepatic (steatosis, cirrhosis) or extrahepatic (lithiasis or biliary obstruction), which means destruction in the kidney’s tissues [48]. 

The toxic effect of ethyl acetate extract, essentially hepatic, characterized by lesion, hepatic necrosis, infiltration, portal and lobular inflammation could be entered in the framework of drug hepatitis compatible with cytolysis syndrome. 

## 4. Materials and Methods

### 4.1. Extraction of Secondary Metabolites

The extraction of secondary metabolites was achieved by cold maceration using methanol-water at 80 % (*v*/*v*). Needles powder was macerated in MeOH-water for 24 h, after which the solvent was evaporated using a rotary evaporator. The crude extract obtained was fractionated using different solvent with increasing polarities, starting with dichloromethane, followed by ethyl acetate (EA) and *n*-butanol (*n*-BuOH). EA and n-BuOH fractions were taken to evaluate their biological activities.

### 4.2. Tyrosinase Inhibitory Ability of Extracts

The tyrosinase inhibition activity was tested according to the method described by [49]. The reaction mixture contains 150 µL of phosphate buffer (pH 6.8), 10 µL of extracts at different concentrations and 20 µL of tyrosinase, the mixture is incubated for 10 min at 37 °C. Subsequently, a volume of 20 μL of the L-DOPA substrate was added. The absorbance was read at 475 nm after 10 min of incubation at 37 °C. Kojic acid was used as a standard for comparison.Each concentration was analyzed in three independent experiments run in triplicate. The inhibitory activity of the tested compounds was expressed as the concentration that inhibited 50% of the enzyme activity (IC_50_). 

### 4.3. Toxicity of Extracts In Vitro, In Vivo and In Silico

#### 4.3.1. In Vitro Cytotoxicity Assay Using Brine Shrimp Lethality Assay

- Hatching Brine Shrimp eggs

Brine shrimp (*Artemia salina*) eggs (JBL Artemio Mix, Germany) were hatched in seawater by mixing 10 g with 1l of seawater. The blend was incubated at 28 °C in an incubator (Panasonic) for 48 h under artificial lighting and aeration provided by an aquarium pump (Champion, Atman^®^). After incubation, the nauplii (larvae) were used in the toxicity assay.

- Brine Shrimp lethality assay

The lethality assay was assessed in a 96-well micro-plate following the method described by [50]. Samples were prepared in distilled water. 100 µL of seawater containing 10 live selected larvae was placed in each well. After that, 80 µL of seawater and 20 µL of the samples at different concentrations were added. The Micro-plate was incubated at 28 °C during 24 h under lighting conditions. Potassium dichromate was used as a standard for comparison. The number of survivors nauplii was counted and the mortality percentages were determined using the following formula:

Mortality (%) = [(Control − Surviving)/Control] × 100

- Control: larvae number in control group (*n* = 10)
- Surviving: surviving larvae number

#### 4.3.2. In Vivo Oral Acute Toxicity Assay

The acute toxicity assay was performed according to the Organisation for Economic Cooperation and Development guideline (OECD) 423. Female *Albino* mice weighing about 20–26 g, having age 8–10 weeks were randomly selected. Mice were divided into three groups; the first group served as control: these mice received distilled water instead of extracts. The second group was treated with ethyl acetate extract, while the third group administered *n*-BuOH extract. The limit test was assessed at 2000 mg/kg, oral administration as a single dose. Mice were kept without food for 3–4 h before gettingextracts, while having access to water ad libitum. The dose was administered to the miceaccording to their body weights. The animal’s behaviors were closely observed for the first 4 h. Food was given after 2–3 h of dosing. All groups were observed closely for any toxic consequences within the first 6 h and then at regular intervals for a total period of 14 days. Surviving mice were observed for the onset of the toxic reactions. Weights of animals were monitored and documented as well. At the end of the experiment, animals were weighed, blood samples were collected by eye orbital sinus route, and serum was separated for biochemical tests. Vital organs (liver and kidneys) were excised after killing mice by cervical dislocation; and were conserved in 10% formaldehyde for histopathological examinations.

All experiments were conducted following US guidelines (NIH publication, 1985) and were approved by the scientific committee of the faculty of Natural and Life Sciences of Mentouri Brothers University, CONSTANTINE 1.

### 4.4. ADMET Properties In Silico 

#### 4.4.1. Identification of Bioactive Molecules in EA and *n*-BuOHFractions

The chemical profile was determined by LC-MS/MS analysis. The LC-MS/MS system used for the quantitative and qualitative analysis of 15 phytochemicals consists of Shimadzu Nexera model UHPLC coupled to Shimadzu LCMS 8040 model triple quadrupole mass spectrometer. The liquid chromatograph is composed of LC-30 AD model gradient pump, DGU-20A3R model degasser, CTO-10ASvp model columnoven and SIL-30AC model autosampler. Chromatographic separation was performed on an Agilent Poroshell 120 model (EC-C18 2.7 µm, 4.6 mm ×150 mm) column. The column temperature was kept at 40 °C during the analysis. The mobile phase consisted of water (A; 5 mM ammonium formate, % 0.15 formic acid) and methanol (B; ammonium formate, % 0.15 formic acid). The applied gradient profile was optimized as 20–100% B (0–25 min), 100% B (25–35 min), 20% B (35–45 min). The flow rate of the mobile phase was 0.5 mL/min and the injectionvolume was 3 µL. The optimum ESI parameters for the mass spectrometer were determined as 350 °C interface temperature, 250 °C DL temperature, 400 °C heat block temperature, and 3 L/min and 15 L/min nebulizer and drying gas (N_2_) flow rates, respectively.

#### 4.4.2. Programs Used and Pharmacokinetic Properties in Silico Study

- PubChem database

The PubChem database has become an important source; it is a bank that contains all the chemical molecules. It provides information on chemical structures, identifiers, chemical and physical properties, biological activities, patents, health, safety, and toxicity data. In the present work, the PubChem database was used to determine the structure and the SMILES of the flavonoids and phenolic compounds identified by LC-MS/MS in the ethyl acetate and *n*-Butanol fractions of the leaves of *Abies numidica* de Lannoy ex Carrière.

- pkCSM Server and pharmacokinetic properties determination.

The pharmacokinetic properties of the leaf extracts of *A. numidica* de Lannoy ex Carrière were determined *in silico* using the pkCSM server and SMILES presented in Table 7. 

### 4.5. Statistical Analysis 

Data were analyzed on Excel software. The results are expressed as mean ± SD. One-way ANOVA test was used. Values at *p* < 0.05 were considered significant.

## 5. Conclusions

Algerian fir is a medicinal plant used in traditional medicine to treat cataplasms and inflammations, but no papers have been published about its toxicity. To the best of our knowledge, this is the first report concerningacute oral toxicity. In this study, the in vitro cytotoxicity of fractions obtained from Algerian fir was investigated against *Artemia salina* larvae, and the acute oral toxicity was also examined. The results indicated the potential cytotoxic effect of both extracts at low concentrations, which indicated the powerful anti-tumour effect of this plant. The fractions revealed no changes occured in neither mice behaviors nor body weights. Acute oral toxicity of both fractions suggested that the LD_50_ was greater than 2000 mg/kg. However, biochemical and histopathological examinations proved hepatic, renal, and lung damage at a dose of 2000 mg/kg. It is very important to conduct other studies in order to thoroughly assess the toxic threshold of this plant. As a result, the toxicological classification will be determined for this endemic plant. To the best of our knowledge, the current research is the first to report the physicochemical, pharmacokinetic, and cytotoxicity effect of molecules determined in *A. numidica* leaves extracts. The recent development of *in silico* approaches helps researchers and scientists to screen and to determine the effective molecules having drug-likeness.

## Figures and Tables

**Table 1:** Behaviors, toxicity signs and body weight change set of mice during the period of treatment.

	30 min		6 h			24 h		48 h		7 days		14 days	
Observation	C	M_tr_	C		M_tr_	C	M_tr_	C	M_tr_	C	M_tr_	C	M_tr_
weight	N	N	N	N	N	N	N	N	N	N	N	N	N
Food consumption	N	N	N	N	N	N	N	N	N	N	N	N	N
Respiration	N	N	N	N	N	N	N	N	N	N	N	N	N
Tremors	A	A	A	A	A	A	A	A	A	A	A	A	A
Pains	A	A	A	A	A	A	A	A	A	A	A	A	A
Convulsions	A	A	A	A	A	A	A	A	A	A	A	A	A
Skin changset	A	A	A	A	A	A	A	A	A	A	A	A	A
Drowsiness	A	A	A	A	A	A	A	A	A	A	A	A	A
Sedation	A	A	A	A	A	A	A	A	A	A	A	A	A
Coma	A	A	A	A	A	A	A	A	A	A	A	A	A
Surviving/death	V	V	V	V	V	V	V	V	V	V	V	V	V

C: Control; M_tr_: treated mice; A: Absent; N: Not observed; V: survival.

**Table 2:** Effect of AE and *n*-BuOH fractions on mice body weight during the test of toxicity.

	Body Weight (g)		
Groups	1st Day	7th Days	14th Days
Contrl	24.00 ± 1.00	28.00 ± 1.73	26.00 ± 2.00
EA (2000 mg/kg)	22.66 ± 1.5	26.00 ± 1.00	26.00 ± 0.00
*n*-BuOH (2000 mg/kg)	25.33 ± 0.94	27.33 ± 1.69	28.66 ± 1.24

**Table 3:** Effect of EA and *n*-BuOH extracts on renal parameters after the toxicity test (* *p* <0.05).

Parameters	Unit	Control	EA (2000 mg/kg)	*n*-BuOH (2000 mg/kg)
Creatinine	mg/L	2.4	3.1 *	4.4 *
Urea	g/L	0.50	0.45	0.43

**Table 4:** Effect of EA and *n*-BuOH extracts on hepatic parameters in mice serum after toxicity test (* *p* < 0.05).

Parameters	Unit	Control	EA	*n*-BuOH
			(2000 mg/kg)	
AST	UI/L	105	137 *	132 *
ALT	UI/L	33	45 *	111 *
Alkaline Phosphatase	UI/L	98	209 *	192 *
Total Bilirubine	mg/L	2	2	2
Direct Bilirubine	mg/L	1	1	1
Gamma-Glutamyl-Transferase	UI/L	2	2	1

ALT: Alanine aminotransferase; AST: aspartate aminotransferase.

**Table 5:** Effect of EA and *n*-BuOH extracts on lipid parameters after the toxicity test.

Parameter	Unit	Control	EA	*n*-BuOH
			(2000 mg/kg)	
Triglycerides	g/L	1.26	1.88	0.86
Total Cholesterol	g/L	1.16	1.05	0.98

**Table 6:** Histopathology of control and extracts (EA, *n*-BuOH) treated groups at a limit dose of (2000 mg/kg).

Organ	T	EA	*n*-BuOH
Liver	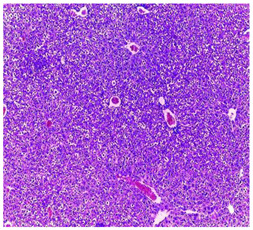	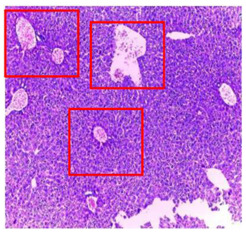	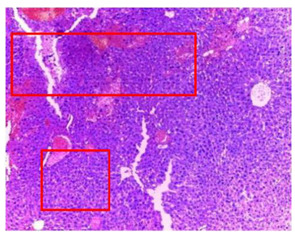
	Hepatic parenchyma with conserved architecture	Vascular congestion, balonization of cytoplasm with clear nucleus, presence of necrosis, acidophilic bodies, basophils, small hyperchromatic nucleus, lobular lymphocyte filtrate.	
Kidneys	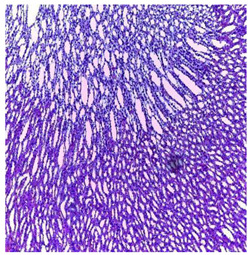	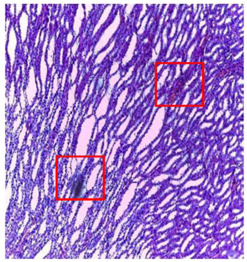	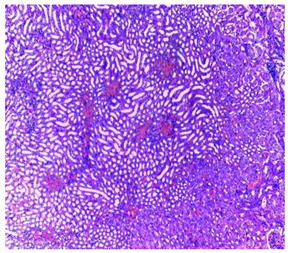
	Round parenchyma surrounded by a fibro-adipose capsule of preserved architecture	Seat of vascular congestion with lesion of interstitial nephritis mainly made up of lymphocytes dispersed between the tubules and by sectors surrounding glomeruli. There was no objective glomerular damage on the limits of the sectors examined. Presence of surrenal gland with conserved morphology and cytology.	
Lung	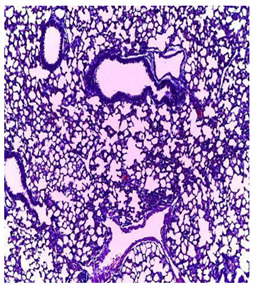	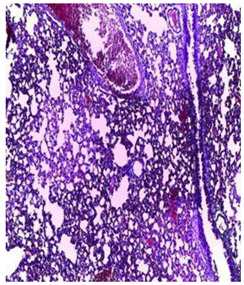	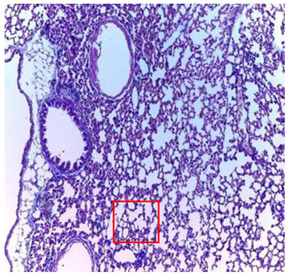
	A pulmonary parenchyma, made of optically empty cavity (alveoli) separated by fibrous septa	Lung parenchyma seat of vascular congestion The vessels have a hyalinised wall with the presence of haemorrhagic diffusion.	Presence of vascular congestion Presence of lymphocytes inflammatory infiltrate

**Table 7:** Structure of molecules determined in EA and *n*-BuOH fractions and their SMILES obtained from Pubchem database.

N°	Analyte	EA (µg/g)	*n*-BuOH (µg/g)	SMILES
1	Hesperidine	7.83	42.02	CC1C(C(C(C(O1)OCC2C(C(C(C(O2)OC3=CC(=C4C(=O)CC(OC4=C3)C5=CC(=C(C=C5)OC)O)O)O)O)O)O)O)O
2	protocatechic acid	71.62	N.D	C1=CC(=C(C=C1C(=O)O)O)O
3	chlorogenic acid	15.59	9.66	C1C(C(C(CC1(C(=O)O)O)OC(=O)C=CC2=CC(=C(C=C2)O)O)O)O
4	Luteolin-7-glucoside	43.17	14.6	C1=CC(=C(C=C1C2=CC(=O)C3=C(C=C(C=C3O2)OC4C(C(C(C(O4)CO)O)O)O)O)O)O
5	Hyperoside	3370.96	399.91	C1=CC(=C(C=C1C2=C(C(=O)C3=C(C=C(C=C3O2)O)O)OC4C(C(C(C(O4)CO)O)O)O)O)O
6	Rutin	27.58	102.62	CC1C(C(C(C(O1)OCC2C(C(C(C(O2)OC3=C(OC4=CC(=CC(=C4C3=O)O)O)C5=CC(=C(C=C5)O)O)O)O)O)O)O)O
7	Apigetrin	192.56	23.19	C1=CC(=CC=C1C2=CC(=O)C3=C(C=C(C=C3O2)OC4C(C(C(C(O4)CO)O)O)O)O)O
8	Quercitrin	2300.33	23.19	C[C@H]1[C@@H]([C@H]([C@H]([C@@H](O1)OC2=C(OC3=CC(=CC(=C3C2=O)O)O)C4=CC(=C(C=C4)O)O)O)O)O
9	Astragaline	3391.36	147.22	C1=CC(=CC=C1C2=C(C(=O)C3=C(C=C(C=C3O2)O)O)O[C@H]4[C@@H]([C@H]([C@@H]([C@H](O4)CO)O)O)O)O
10	Quercetin	24.75	N.D	C1=CC(=C(C=C1C2=C(C(=O)C3=C(C=C(C=C3O2)O)O)O)O)O
11	Luteolin	2.41	N.D	C1=CC(=C(C=C1C2=CC(=O)C3=C(C=C(C=C3O2)O)O)O)O
12	Apigenin	6.64	N.D	C1=CC(=CC=C1C2=CC(=O)C3=C(C=C(C=C3O2)O)O)O

**Table 8:** In silico pharmacokinetic properties of phenolic compounds from the leaves of *A. numidica* de Lannoy ex Carrière.

**a: Pharmacokinetic Properties**										
**N**	**Analyte**	**Lipinski Rules**					**Absorption**		**Distribution**	
		**MW (g/mol)**	**log P**	**RT**	**A**	**D**	**Intest (%)**	**Skin (log Kp)**	**DV (log L/kg)**	**BBB (log BB)**
1	Hesperidin	610.565	−1.1566	7	15	8	31.481	−2.735	0.996	−1.715
2	Protocatechic acid	154.121	0.796	1	3	3	71.174	−2.727	−1.298	−0.683
3	chlorogenic acid	354.31	−0.6459	4	8	6	36.377	−2.735	0.581	−1.407
4	Luteolin-7-glucoside	448.38	−0.2445	4	11	7	37.556	−2.735	0.884	−1.564
5	Hyperoside	464.379	−0.5389	4	12	8	37.556		0.884	−1.564
6	Rutin	610.521	−1.6871	6	16	10	23.446		1.663	−1.899
7	Apigetrin	432.381	0.0499	4	10	6	37.609		0.342	−1.391
8	Quercitrin	448.38	0.4887	3	11	7	52.709		1.517	−1.495
9	Astragalin	448.38	−0.2445	4	11	7	48.052		1.444	−1.514
10	Quercetin	302.238	1.988	1	7	5	77.207		1.559	−1.098
11	Luteolin	286.239	2.2824	1	6	4	81.13		1.153	−0.907
12	Apigenin	270.24	2.5768	1	5	3	93.25		0.822	−0.734
**b: Pharmacokinetic Properties**										
**N**	**Analyte**	**Metabolsim**					**Clearance**		**Toxicity**	
		**P450 (Yes/No)**		**P450** **Substrats**			**TC**	**Subs. OCT2 (Yes/No)**	**Hepatotoxicity (Yes/No)**	**MTD**
1	Hesperidine	No		No			0.211	No	No	0.525
2	protocatechic acid	No		No			0.551			0.814
3	chlorogenic acid	No		No			0.307			−0.134
4	Luteolin-7-glucoside	No		No			0.478			0.584
5	Hyperoside	No		No			0.394			0.569
6	Rutin	No		No			−0.369			0.452
7	Apigetrin	No		No			0.547			0.515
8	Quercitrin	No		No			0.364			0.495
9	Astragalin	No		No			0.462			0.582
10	Quercetin	Inhi. CYP1A2		No			0.407			0.499
11	Luteolin	Inhi. CYP1A2		No			0.495			0.499
12	Apigenin	Inhi. CYP1A2 and CYP2C19		No			0.566			0.328

MW: molecular weight; RT: Rotary connections; A: acceptors; D: Donors; Intest: Intestinal; DV: Distribution volume; BBB: Brain Blood Barrier. TC: Clearance totale; - P450: inhibitors of cytochrome 450; MTD: Maximum tolerated dose.

## Data Availability

Not applicable.
